# Word Processing Is Faster than Picture Processing in Alzheimer's Disease

**DOI:** 10.1155/2020/9541869

**Published:** 2020-01-25

**Authors:** Kenichi Meguro, Yumi Takahashi, Masahiro Nakatsuka, Jiro Oonuma, Keiichi Kumai, Mari Kasai, Satoshi Yamaguchi

**Affiliations:** Geriatric Behavioral Neurology Project, Tohoku University New Industry Hatchery Center (NICHe), 4-1, Seiryo-machi, Aoba-ku, IDAC, 980-8575 Sendai, Japan

## Abstract

**Objective:**

Alzheimer's disease (AD) is characterized by a slow progressive impairment of episodic memory. Many studies have shown that AD exhibits deterioration of semantic memory during the course of disease progression. We previously reported that AD patients exhibited severe access disorders in the semantic memory system, using the Momentary Presentation Task (20 or 300 ms). In this study, we studied access disorder in patients with AD by the use of object difference (pictures vs words) methods.

**Methods:**

56 patients with probable AD (NINCDS-ADRDA, mean age 79.0 years) and 11 healthy controls (HC) (mean age 67.0 years) were studied. Ten pictures and 10 corresponding Japanese Hiragana words were presented arbitrarily for 20 and 300 ms on the monitor screen which were correctly named at the usual confrontation setting (i.e., semantic memory preserved). They were asked to name the pictures or to read the words or nonsense syllables aloud.

**Results:**

The AD group showed significantly lower scores than the HC group, especially for the 20 ms condition. For the type of stimuli, the AD patients had better performances for words > pictures > nonsense syllables, although no differences for the HC group. The effect of AD severity was noted, moderate > severe stage.

**Conclusions:**

Our results suggested that the processing speed in AD patients may have reduced, even if the semantic memory were preserved. These data indicated that the difference in the processing speeds by the type of stimuli (pictures, words, and nonsense syllables) may be a character of AD patients.

## 1. Introduction

Alzheimer's disease (AD) is characterized by a slow progressive impairment of episodic memory. Many studies have shown that AD exhibits deterioration of semantic memory during the course of disease progression. Semantic memory is the aspect of human memory that corresponds to general knowledge of objects, meaning of words, facts, and people without connection to any particular time or place [1]. Those memories have a different neural basis than episodic memory.

Giffard et al. [2] reported that the AD patient's concrete words are remembered better than abstract words. Some studies, using the naming task, the Semantic Association Task, and the Semantic Knowledge Task, showed that the score of living stimuli was more impaired than the score of nonliving stimuli [3, 4]. Small and Sandhu [5, 6] reported that naming ability was affected relative to the frequency of item use. As shown above, there are many previous studies that focused on the effect of the category of words or objects on semantic memory impairment.

In order to understand the psychological and neural basis of semantic memory impairment, many researchers have proposed various pathological models in which “semantic memory network” is affected. Yang et al. [7] suggested that loss of semantic structure and an inability to access semantic knowledge occur in the pathogenesis of AD. Passafiume et al. [8] suggested a breakdown of the semantic network rather than a deficit in the access to the semantic store. Hodges et al. [9] indicated that AD patients consistently perform poorly across different semantic tasks identifying the same item and argued for semantic storage degradation. The nature of the cognitive dysfunction responsible for these impairments is still a matter of controversy.

In our study [10], we compared semantic dementia (SD) with AD in deficits of semantic memory. We hypothesized that impaired semantic access with preserved semantic memory would be reflected in successful performance on those tasks without a time restriction but impaired performance with a time restriction. We thus defined “the access deficit” as follows: the decreased performances of participants in spite of preserved items in semantic memory, in both picture naming and word reading, using the “Task with Momentary Presentation” (restriction of the presentation time: 20 ms and 300 ms). In this case, we defined “items preserved semantic memory” as follows: participants could name pictures and read words correctly and also could make correct answers in two-way matching (picture-to-word and word-to-picture), without time restriction. We reported that the access deficit occurred in both SD patients and AD patients, and this deficit is more severe in SD patients. Our data indicated that patients with AD have impairment of access of visual pathways.

Rizzo et al. [11] reported that patients with AD performed significantly worse than participants without dementia on tests of static spatial contrast sensitivity, visual attention, shape-from-motion, color, visuospatial construction, and visual memory. They suggested that visual dysfunction in AD may contribute to performance decrements in other cognitive domains [11].

The visual dysfunction in patients with AD may include the impaired visual processing speed and the semantic system. We assumed the following hypothesis:

1. The deficits of visual processing speed in AD patients may affect the semantic system, rather than HC
2. In the processing function of the semantic system, the degree of disability may depend on the types of stimuli (pictures, words, and nonsense syllables) in AD patients
3. The impairment of visual processing speed may depend on the severity of AD

The purpose of this study was to detect the difference of disorders of the information processing of pictures, words, and nonsense syllables in between patients with AD and HC participants. We focused on the processing speed and performed a visual presentation task in the presentation time (20 ms-300 ms) of visual stimuli (pictures or words) for patients with AD and HC participants. This is the first report of the processing speed of pictures, words, and nonsense syllables in AD patients.

## 2. Methods

### 2.1. Participants

Fifty-six outpatients with AD were recruited from the Tajiri Clinic, an integrated institute for stroke and dementia, which is situated in Osaki, Miyagi Prefecture, in northern Japan. Patients with AD who were seen at the Tajiri Clinic from February 2011 to June 2014 were enrolled. All patients underwent routine medical examinations, including head magnetic resonance imaging (MRI), chest X-ray, electrocardiogram, laboratory urine and blood tests, and neuropsychological examinations.

The sole entry criterion was probable AD according to the National Institute of Neurological and Communicative Disorders and Stroke-Alzheimer's Disease and Related Disorders Association (NINCDS-ADRDA) [12].

The exclusion criteria for this study were as follows: (1) other neurological, psychiatric, or systematic diseases (e.g., stroke, depression, and alcoholism); (2) clinically notable MRI (e.g., stroke lesions); (3) low vision as defined by the World Health Organization (visual acuity or corrected visual acuity is less than 0.3); and (4) severe AD patients whose understanding of instruction is difficult (in an operational way, Mini-Mental State Examination (MMSE) [13] scores lower than 7 at the evaluation).

### 2.2. Healthy Control

Eleven healthy control participants were recruited from the Sendai Medical Imaging Clinic, which is located in Sendai, Miyagi Prefecture, in northern Japan. Healthy controls (HC) underwent MRI, FDG-PET imaging, blood tests, and neuropsychological examinations and were proved to be free of cognitive dysfunction and history of cerebrovascular disease.

The exclusion criteria were as follows:

1. Other neurological, psychiatric, or systematic diseases (e.g., stroke, depression, and alcoholism)
2. Clinically notable MRI findings (e.g., stroke lesions)
3. Low vision as defined by the World Health Organization (visual acuity or corrected visual acuity was less than 0.3)
4. MMSE scores lower than 25 at the evaluation

### 2.3. Visual Acuity Test

All the participants' visual acuity (distance, 5 m) and near vision (distance, 0.3 m) were tested using the Landolt ring test. We confirmed that each subject's visual acuity was intact in this study.

### 2.4. Cognitive Function Tests

We used the MMSE for assessing global cognitive function for all participants.

We used the Trail Making Test (TMT-A) [14] as a visual attention test and the digit symbol of Wechsler Adult Intelligence Scale-III (WAIS-III) [15] as an executive function test for all participants.

### 2.5. Task with Momentary Presentation

The Task with Momentary Presentation is our original task to examine a processing speed of visual information [10].

The pictures used in the picture classification task in the standard higher visual perception test present only inanimate objects. Therefore, we prepared 10 photographs including those of natural items to avoid a categorical bias. To unify the speech processing speed, we selected 10 natural and 10 inanimate objects that can be expressed with 3 Hiragana letters and selected 5 items for which the patient was able to do a two-way matching: photograph-to-word and word-to-photograph matching. The tasks were prepared using computer software (MATLAB, MATrix LABoratory) and displayed for 20 or 300 msec on a 14-inch CRT monitor (Cathode Ray Tube). The letter size was 1.3 × 1.3 cm.

Regarding understanding of instructions in momentary presentation and visual recognition of the display, a single Hiragana letter was presented for 20 msec, and the patient read it aloud. The abilities of the patients to distinguish shapes and to read aloud and their understanding of the test procedure were confirmed before the test was performed. We did not compare Hiragana and Chinese characters, since the latter is difficult to test in patients with dementia.

To complete the photograph task, the patient named the photograph (naming) and pointed at one card indicating the photograph out of 4 Hiragana word cards (pointing). For the word task, the patient read the word aloud and pointed at one photograph identifying the word out of 4 photograph cards.

## 3. Analyses

### 3.1. Comparison of the Performance of Task with Momentary Presentation in the AD Group and the HC Group

We compared AD with HC participants (the total number of correct responses of the Task with Momentary Presentation: pictures, words, and nonsense syllables) in the performances of 2 presentation times (20 ms vs 300 ms). We used the two-way repeated ANOVA (2 groups; AD vs HC × 2 presentation times; 20 ms vs 300 ms) with the covariate's age and education for the total number of correct responses of the Task with Momentary Presentation.

### 3.2. Effect of the Type of Stimuli: Pictures, Words, or Nonsense Syllables—AD vs NC

In the AD and HC groups, we analyzed the performance of Momentary Presentation Task in 3 types of stimuli (pictures, words, or nonsense syllables) of 2 presentation times (20 ms vs 300 ms). We used the two-way repeated ANOVA (3 stimuli; pictures vs words vs nonsense syllables × 2 presentation times; 20 ms vs 300 ms) for the total number of correct responses of the Momentary Presentation Task.

### 3.3. Effect of the Severity of AD

Since the median of MMSE score was 17 in AD patients in this study, we divided AD patients into two groups operationally: the mild AD group (MMSE score ≥ 17) and the moderate AD group (MMSE score < 17). We used the two-way repeated ANOVA (2 groups; mild vs moderate × 2 presentation times; 20 ms vs 300 ms) for the total number of correct responses of the Task with Momentary Presentation.

### 3.4. Error Pattern Analysis in AD Patients

In pictures, three responses including correct and two error patterns were classified as follows: (1) correct response; (2) semantic-type error: incorrect response on similar color or shape object (for example, in the case of “shears” as a correct response, the subject named it “knife” incorrectly); and (3) other-type error: other-type incorrect response but not a semantic error.

In words, three responses including correct and two error patterns were classified as follows: (1) correct response; (2) semantic-type error: incorrect response on similar spelling of actual word (for example, in the case of “Ha-Sa-Mi (shears)” as a correct response, the subject misread it as “Ha-Sa-Mu (sandwich)”; and (3) other-type error: other-type incorrect response but not a semantic error.

In nonsense syllables, three responses including correct and two error patterns were classified as follows: (1) correct response; (2) semantic-type error: incorrect response on similar spelling of nonexistent word (for example, in the case of “Ha-Mi-Sa (nonsense syllables)” as a correct response, the subject misread it as “Ha-Sa-Mi (shears)”; and (3) other-type error: other-type incorrect response but not a semantic error.

The number of reactions of the Task with Momentary Presentation each of 20 ms and 300 ms was analyzed by Chi-square analysis for every reaction type (correct, semantic-type error, and other-type error).

### 3.5. Effect of Cholinesterase Inhibitor (ChEI) Medication

We compared the total number of correct answers of the Task with Momentary Presentation: pictures, words, and nonsense syllables between the ChEI medication group (*n* = 47) and the non-ChEI medication group (*n* = 9). We used the two-way repeated ANOVA (2 groups; ChEI vs non − ChEI × 2 presentation times; 20 ms vs 300 ms) with the covariate's MMSE scores and education.

### 3.6. Ethics

Written informed consent was obtained from all participants and from the family of those with dementia. This study was approved by the Ethics Committee of Tohoku University Graduate School of Medicine.

## 4. Results

### 4.1. Participants

Demographics for the two groups are shown in Table 1. The AD group was significantly older than the HC group. In education and near vision, the AD group was significantly lower than the HC group.

### 4.2. Analysis 1: Comparison of the Performances of Momentary Presentation Task in the AD and HC Groups

**Figure 1:**
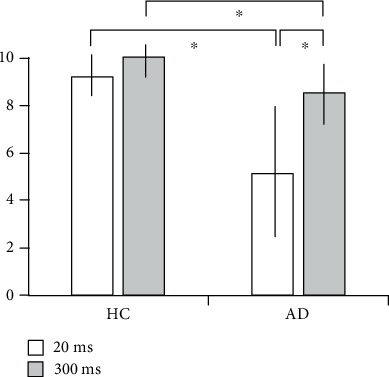
Comparison of the performances of the two groups. AD = Alzheimer's disease; HC = healthy control. ^∗^*p* < 0.005, post hoc test. The *y*-axis represents the average number of correct answers with SD. According to the two-way ANOVA with time as the repeated measure and with age and education as covariables, there was a significant group effect (*F* = 11.6, *p* = 0.001) and a presentation time effect (*F* = 6.3, *p* = 0.013), with a significant interaction (*F* = 13.1, *p* < 0.001). There was a significant effect on the covariate for age (*F* = 12.7, *p* < 0.001), but not for education (*F* = 1.9, *p* = 0.173). A post hoc *t*-test showed that the AD group had a significantly lower score than the HC group in presentation time for both 20 ms (*t* = −6.77, *p* < 0.001) and 300 ms (*t* = −3.58, *p* < 0.001). A post hoc *t*-test in the AD group showed that the presentation time for 20 ms has a significantly lower score than that for 300 ms (*t* = −17.26, *p* < 0.001).

Figure 1 illustrates the results of 20 ms and 300 ms of the two groups. The AD group had lower scores than the HC group in both presentation times.

### 4.3. Analysis 2: Effect of the Type of Stimuli in the HC and AD Groups

**Figure 2:**
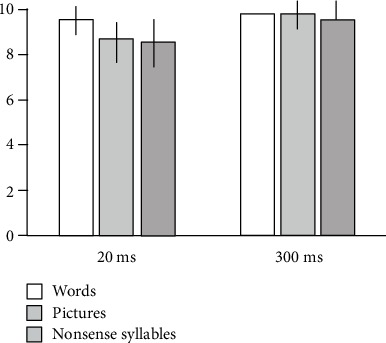
Effect of the types of stimuli in the HC group. HC = healthy control. The *y*-axis represents the average number of correct answers with SD. There was no significant difference among the three types of stimuli (pictures, words, and nonsense syllables) in the two-way ANOVA with repeated measures (*F* = 1.08, *p* = 0.359), with the presentation time effect (*F* = 9.52, *p* = 0.012), and with no interaction (*F* = 1.32, *p* = 0.285).

**Figure 3:**
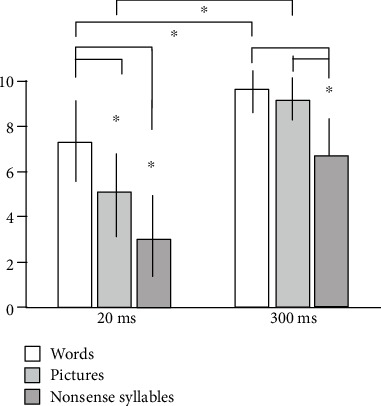
Effect of the types of stimuli in the AD group. AD = Alzheimer's disease. ^∗^*p* < 0.001, post hoc. The *y*-axis represents the average number of correct responses with SD. There was a significant effect of the type of stimuli (*F* = 89.2, *p* < 0.001) and the presentation time effect (*F* = 192.1, *p* < 0.001) with a significant interaction (*F* = 9.1, *p* < 0.001) using the two-way ANOVA with repeated measures. From the paired *t*-test as a post hoc test in 20 ms, the picture's scores were significantly lower than word's scores (*t* = −6.89, *p* < 0.001), and nonsense syllables' scores were significantly lower than word's scores (*t* = −11.54, *p* < 0.001). Nonsense syllables' scores were significantly lower than picture's scores (*t* = −4.68, *p* < 0.001), with a correct response. From the paired *t*-test as a post hoc test in 300 ms, nonsense syllables' scores were significantly less than word's scores (*t* = −8.93, *p* < 0.001), and nonsense syllables' scores were significantly less than picture's scores (*t* = −7.88, *p* < 0.001).

Figure 2 illustrates the total number of correct responses (pictures, words, and nonsense syllables) of the HC groups. There was no significant difference among the three types of stimuli.  Figure 3 demonstrates those of the AD group. They have better performances for words > pictures > nonsense syllables.

### 4.4. Analysis 3: Effect of the Severity of AD

The moderate AD group had significantly poorer scores than the mild AD group. There was a significant group effect in a two-way ANOVA with time as the repeated measure (between the mild group and the moderate group; *F* = 11.08, *p* = 0.001). The presentation time had a significant effect (*F* = 296.5, *p* < 0.001). The interaction between the severity of AD and the presentation time was not significant (*F* = 0.41, *p* = 0.524).

### 4.5. Analysis 4: Error Pattern Analysis in AD Patients

**Figure 4:**
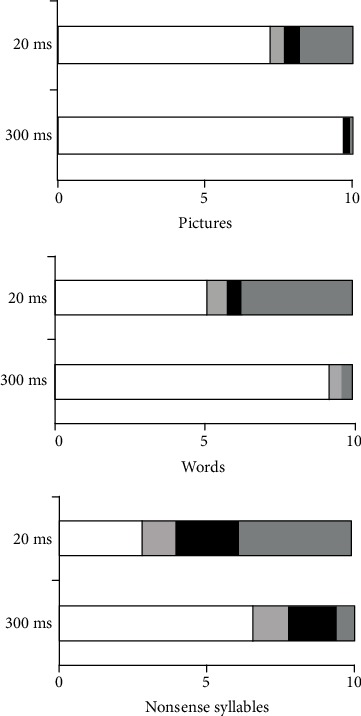
Error patterns in the AD group. AD = Alzheimer's disease. We classified the error patterns into 4 types (see the text). The presentation times are shown on the *y*-axis and the total numbers of responses are shown on the *x*-axis. There were significant differences between the pattern of responses and the presentation time in pictures (*χ*^2^ = 239, *p* < 0.001), words (*χ*^2^ = 129, *p* < 0.001), and nonsense syllables (*χ*^2^ = 157, *p* < 0.001).

Figure 4 shows the total number of different response types in each of the three types of stimuli in both two presentation times of responses.

#### 4.5.1. Pictures (Figure 4(a))

In the 20 ms presentation time, the number of the semantic-type error was less than that of the other-type error. In the 300 ms presentation time, the number of the semantic-type error was slightly decreased; however, the other-type error was markedly decreased.

#### 4.5.2. Words (Figure 4(b))

In the 20 ms presentation time, the number of the semantic-type error was less than that of the other-type error. In the 300 ms presentation time, there was no semantic-type error pattern, and the other-type error was markedly decreased.

#### 4.5.3. Nonsense Syllables (Figure 4(c))

In the 20 ms presentation time, the number of the semantic-type error was less than that of the other-type error. In the 300 ms presentation time, there were several semantic-type errors as in 20 ms, and the other-type error was markedly decreased.

### 4.6. Analysis 5: Effect of Cholinesterase Inhibitor (ChEI) Medication

There was no significant difference in age (*t* = −0.55, *p* = 0.584), gender (*X*^2^ = 1.07, *p* > 0.1), or near vision (*t* = 0.73, *p* = 0.468) between the ChEI group and the non-ChEI group. The non-ChEI group was significantly higher than the ChEI group in terms of MMSE scores (*t* = 2.47, *p* = 0.014) and education (*t* = 2.85, *p* = 0.005). According to the two-way ANOVA with repeated measure and with MMSE scores and education as covariables, there was no significant difference between the ChEI group and the non-ChEI group (*F* = 0.46, *p* = 0.500).

## 5. Discussion

Visual processing and semantic processing are distinct functions, although there might be potential effects of an impairment of visual processing on semantic processing. We herein concentrate on the “visual processing” matter.

### 5.1. Information Processing of AD

Our results revealed that the processing speed of both words and pictures of the AD group was slower than those of the HC group. In the AD group, the deficits of the processing speed depended on the types of stimuli (pictures, words, and nonsense syllables). In contrast, there was no significant difference among the types of stimuli in the NC group. Decreased visual processing speed in the AD group depended on the degree of disease. There was a difference in error pattern on the Task with Momentary Presentation among the three patterns on the types of stimuli (pictures, words, and nonsense syllables) in AD patients. Our results showed that the AD group required a longer time for recognizing “watching visual stimuli” than the HC group. These results showed that the processing speed of visual information of letter was delayed in AD, which supported Bublak's study using vertically arranged five letters of alphabet (Bublak et al.) [16]. In this study, we used the visual tasks including words, nonsense syllables, and pictures to preserve semantic memory. We found that the processing speed in AD patients may have reduced, even if the semantic memory were preserved.

In AD patients, there was a difference in the processing speed among the three patterns of the types of stimuli. Especially, the processing speed of nonsense syllables was severely reduced than other types of stimuli. Previously, there was no study of the relationships between the processing speed and the type of stimuli in AD patients. Our study indicated that the difference in the processing speed by the type of stimuli (pictures, words, and nonsense syllables) may be a character of AD patients. These findings suggest that each processing of object including pictures, words, or nonsense syllables may have a different neural base.

### 5.2. Hypothesis of Cognitive Route of Visual Word and Picture in AD

**Figure 5:**
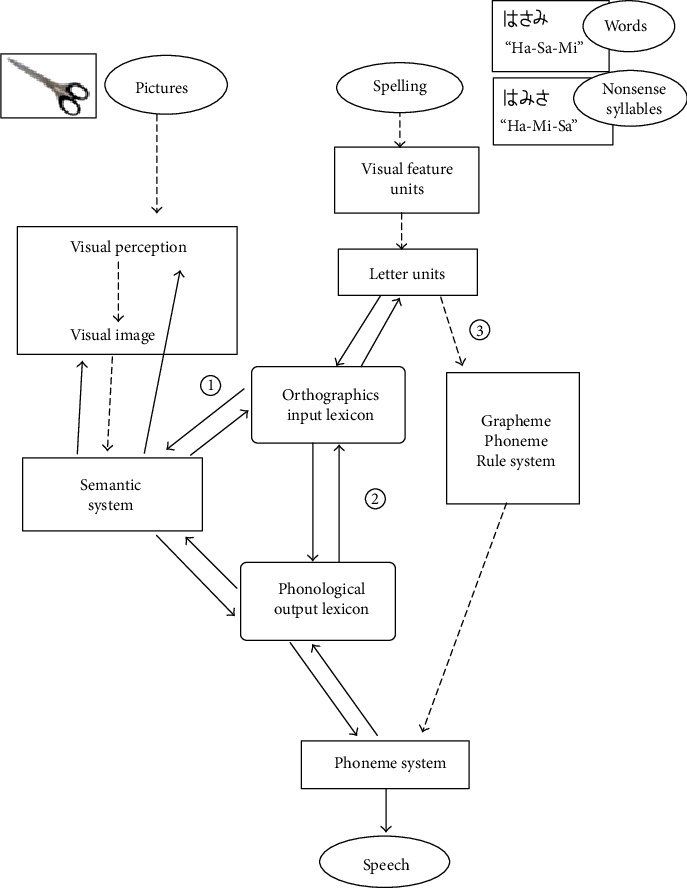
Hypothesis of cognitive route of visual spellings and pictures in AD. AD = Alzheimer's disease. We showed our hypothesis of the information processing for visual spelling and photos in AD patients. The dashed lines indicate the decreased processing function. In this figure, routes (1) to (3) show the dual-route cascaded model of visual word recognition and reading aloud. Word processing may be faster than nonsense syllables and pictures, because the excitatory connection in word processing from “orthographic input lexicon” may work normally. Nonsense syllables' processing, in the case of 300 ms, may be slower than word processing, because “Grapheme-Phoneme Rule (GRS) System” may not have an excitatory connection from “Phoneme System.” When AD patients saw the multiple components of characters, they might process them as “meaningful” words (for example, in the case of “Ha-Mi-Sa (nonsense syllable)” as a correct answer, the participant misread it as “Ha-Sa-Mi (shears)”). In our hypothesis, since pictures contain the amount of visual information rather than words, AD patients need to take more time than healthy controls in the process of forming the morphological image by integrating them. During the short presentation time, the connection of the particular visual information and the semantic system in an incomplete processing may lead to the selection of the wrong answer in the naming task (for example, in the case of “shears” as a correct answer, the subject named it “knife” incorrectly).

Figure 5 The hypothesis of cognitive route of visual word and picture in AD.

Figure 5 shows the hypothesis of cognitive route of visual and picture in AD.

The cognitive route of visual word consists of three cognitive routes: (1) the lexical semantic route, (2) the lexical nonsemantic route, (3) and the grapheme-phoneme corresponded (GPC) route. Coltheart et al. [17] described that the activation of GPC route occurs unidirectionally. However, other lexical routes connect bidirectionally. In the case of the word processing, since both the semantic system (route (1)) and the input lexicon (route (2)) work, the processing of words is able to work faster than the processing of nonsense syllables under the condition of the dysfunction of Grapheme-Phoneme Rule System (route (3)).

Japanese Hiragana is a phonogram without the meaning, so the orthographic input lexicon and the phonological output lexicon are a 1 to 1 correspondence, so words can be processed and error of semantic type reduced. In the case of AD patients, when the presentation time was extended to 300 ms, the semantic-type errors did not decrease. When AD patients saw the multiple components of characters, they might process them as “meaningful” words.

We studied the function of naming and reading aloud in AD patients and semantic dementia (SD) patients using the rapid presentation task [10]. The current study and our previous research [10] propose a hypothesis that the processing speed of the word is faster than nonsense syllables working with the lexical nonsemantic route in AD patients. The performance of AD patients improved in the presentation time of 300 ms, comparing the presentation time of 20 ms; however, the performance of SD patients had a slight improvement in the presentation time of 300 ms. We thought that the pathway from the lexicon did not work normally in the SD patient by extending the presentation time, because the SD patient had an impairment of the semantic system.

Patterson et al. [18] reported of the neural basis of semantic memory disorder in SD and AD patients. They described that SD patients had a regional atrophy of the temporal pole and adjacent rostral-inferior surface, but AD patients had widely atypical degenerative lesions. For this neuropathology, we have a hypothesis that the essence of semantic memory disorder of AD patients may be the failure of the processing function.

Why was the picture processing lower than the word processing in AD patients in this study? The recognition of pictures may consist of several paradigmatic processes, from perception of elements to morphological image and object decision in stages. As our hypothesis, since pictures contain the amount of visual information rather than words, AD patients need to take more time than healthy controls in the process of forming the morphological image by integrating them. Marsh and Hillis [19] indicated that the individual components of picture could access the individual semantic functions. In a short presentation time, the connection of the particular visual information and the semantic system in an incomplete processing step may lead to the selection of the wrong answer in the naming task. Therefore, the picture processing was slower than the word processing in the presentation time of 20 ms; however, the results of the picture processing were close to the word processing in the presentation time of 300 ms.

In this study, the severity of AD influenced the processing speed of words, nonsense syllables, and pictures. These findings are consistent with the results of Bublak et al. [16] that their tasks of the processing speed related to the MMSE scores and the CDR stages. Our results suggested that the processing speed on the semantic system decreased with the progressive course of AD.

Our results indicated that the medication using ChEI could not improve the processing speed, which was disaccord with the report of Bublak et al. [16] that nonmedicated patients had a slow processing speed compared with ChEI-medicated patients. As an indicator of processing speed, Bublak et al. [16] used the recognized number of characters per second. Bublak et al. [16] reported that nonmedicated patients also had a decrease in capacity of visual short-term memory (VSTM) storage compared to medicated patients, but not a statistically significant reduction. We thought that there were two factors of processing speed and visual short-term memory capacity per second. To measure a separate processing speed, we used a word consisted of three Hiragana letters so that AD patients could get to a correct response in the study of Bublak et al. [16]. The ChEI effect described in the study of Bublak et al. might relate to visual attention. We compared the results of nonsense syllables between medicated patients and nonmedicated patients. However, there was no significant difference between the two groups.

### 5.3. Limitations

In this study, we used 10 items of objects which remained in the semantic memory in AD patients to focus the disturbance of processing speed. These 10 items were daily high-frequency objects including 3 living and 7 manmade items. We did not focus on the difference by the category of living and manmade items in this study.

All our picture tasks were photos of only meaningful and existent objects. We made consideration of examination time and subject's fatigue; we used three types of stimuli (picture, word, and nonsense syllables). In the future, we need to prepare several nonsense pictures to compare with existent objects. In AD patients, the scores and SD values of our tasks vary widely. The variation in the performance of AD patients did not relate the severity of cognitive dysfunction in those patients. Although our AD patients had no severe Balint-like visual inattention as a result of typical posterior cortical atrophy [20], we could not exclude the patients suffering from atypical atrophy.

## Figures and Tables

**Table 1:** Demographics for the two groups.

	AD *n* = 56	HC *n* = 11	*p* value (*t*-test)
Age	78.8 (6.2)	67.9 (10.7)	0.003
Gender (M/W)	16/40	6/5	
Education	9.7 (2.4)	12.7 (2.0)	0.001
MMSE	17.4 (5.3)	28.0 (2.0)	0.001
V.A.	0.4 (0.2)	0.7 (0.2)	0.02

AD = Alzheimer's disease; HC = healthy control; M = men; W = women; MMSE = Mini-Mental State Examination; V.A. = visual acuity. There were significant differences in age, education, and near vision between the groups (*p* < 0.05).

## Data Availability

The data used to support the findings of this study are restricted by the Ethical Committee of Tohoku University in order to protect patient's privacy. Data are available from Kenichi Meguro for researchers who meet the criteria for access to confidential data.
